# Individual and community-level factors associated with early initiation of antenatal care: Multilevel modelling of 2018 Cameroon Demographic and Health Survey

**DOI:** 10.1371/journal.pone.0266594

**Published:** 2022-04-06

**Authors:** Francis Appiah

**Affiliations:** 1 Berekum College of Education, Berekum, Ghana; 2 School of Public Health, Kwame Nkrumah University of Science and Technology, Kumasi, Ghana; 3 Department of Population and Health, University of Cape Coast, Cape Coast, Ghana; Wollega University, ETHIOPIA

## Abstract

**Background:**

Early initiation of antenatal care (ANC) provided by skilled personnel is essential as it enables pregnant women to receive comprehensive reproductive health services. Early ANC utilisation could prevent complications related to pregnancy and improve maternal and neonatal health outcomes. Regardless of this, only forty-one in every hundred women in Cameroon seek early ANC services. Studies on the uptake of antenatal care in Cameroon have not focused on individual and community-level factors that influence early initiation of ANC. This study aimed at investigating the association between individual and community-level factors and early ANC uptake in Cameroon.

**Methods:**

This study was a cross-sectional survey design. Data was extracted from the women’s file of the 2018 Cameroon Demographic and Health Survey (CDHS). A sample of 4,183 women aged 15–49 who had complete information on variables of interest to the study was used. The outcome variable was early ANC (i.e. women whose first ANC occurred between 0–3 months of pregnancy). Eighteen explanatory variables consisting of fifteen individual-level variables and three community-level variables were selected for the study. At 95% confidence interval (95% CI), two-level multilevel models were built. The results for the fixed effects were presented in adjusted odds ratio (aOR) and the random effects were expressed in terms of Intra-Class Correlation (ICC) and Primary Sampling Units (PSUs) variance.

**Results:**

Descriptively, 46% [CI = 45.0–48.0] of the women aged 15–49 attended ANC earlier. The median age at which women started utilising early ANC was 28 (15 to 48) years. For the fixed effects results, it was found that the odds of seeking early ANC increased among those aged 35–39 [aOR = 1.78, CI = 1.24–2.57], the richest [aOR = 2.43, CI = 1.63–3.64] and those with secondary/higher education [aOR = 1.38, CI = 1.05–1.82]. Muslims [aOR = 0.73, CI = 0.60–0.88] and women at parity four or more had lesser odds to seek early ANC [aOR = 0.63, CI = 0.49–0.82]. The study found that primary sampling unit (community/cluster) [σ^2^ = 0.53, CI = 0.40–0.72] and individual [σ^2^ = 0.16, CI = 0.09–0.29] level variations exist in early initiation of ANC. About 14% (intra-class correlation (ICC) = 0.14) and 5% (ICC = 0.05) variability in early initiation of ANC were attributable to variations in the primary sampling unit (community/cluster) and individual-level factors, respectively.

**Conclusion:**

Individual-level factors (maternal age, wealth status, educational attainment and religious affiliation) were associated with early initiation of ANC whereas variations in cluster/community characteristics contributed to the variations in early initiation of ANC seeking. The Departments of Health Promotion, Health Information Center and eHealth under the Ministry of Public Health, Cameroon, have to strengthen mass sensitisation programs on early ANC uptake and such programs should consider individual differences such as age, wealth status, education, and religious affiliation in its program design.

## Background

The sustainable development goal three (SDG 3), target one was set to reduce the global maternal mortality ratio to less than 70 per 100,000 livebirths by the end of 2030 [1]. To achieve this, a strong political commitment from countries especially those with poor maternal health outcomes, to control their preventable maternal morbidities and mortality are required [2–4]. As a result, the World Health Organisation (WHO) recommends that every woman should seek antenatal care (ANC) from a skilled provider. Following this recommendation, women should have their first ANC visit immediately after conception to twelve weeks of gestation–early ANC and extra seven visits be made after twelve weeks of gestation through to delivery [4]. Early ANC provided by skilled personnel is essential as it enables pregnant women to receive varied and comprehensive reproductive health services. These services include accurate assessment of gestational age to allow for precise treatment of preterm labour, screening for disorders, provision of folic acid supplements, and screening as well as treatment for iron deficiency anaemia and sexually transmitted infections [4–7]. Early ANC could offer health staff the opportunity to meet pregnant mothers and provide them guidance targeted at modifying risky behaviours such as smoking, alcohol consumption, drug abuse, obesity, malnutrition, and occupational exposures [4, 7].

Global report on early ANC indicates an increase in early ANC coverage from 40.9% in 1990 to 58.6% in 2013 [4]. However, disparities in the uptake of early ANC exist based on geographical locations. Whereas high-income countries recorded 84.8% of early ANC coverage in 2013, low-income countries’ early ANC coverage stalled at 48.1% [4]. Sub-Saharan Africa (24.9%) is among the notable regions which recorded the lowest early ANC visit coverage, globally [4]. Cameroon has seen a marginal rise in early ANC attendance from 33% to 35% between 1991 and 2014 [8]. However, the 2018 Cameroon Demographic and Health Survey (CDHS) reported that only forty-one in every hundred women (41%) in Cameroon sought early ANC services [8]. This implies that most women in Cameroon might not fully benefit from treatment and preventive measures covered under early ANC services such as the provision of iron and folate supplements for the treatment of anaemia, administering Intermittent Preventive Treatment (IPTp) to curb malaria in pregnancy, immunization against tetanus, tuberculosis, nutrition, and detection and management of HIV/AIDS and other sexually transmitted infections (STIs) [4–7].

Apart from the low uptake of early ANC in Cameroon, several studies have been done on predictors of ANC attendance in Cameroon [9–12]. However, none of them have considered individual and community-level factors that influence early ANC uptake. For instance, Halle-Ekane et al. [9] assessed the content and utilization of ANC services in a rural community in Cameroon whereas Halle-Ekane et al. [10] focused on the quality of antenatal care and outcome of pregnancy in a semi-urban area in Fako Division. The study of Tolefac et al. [11] also investigated why pregnant women present late for their first antenatal care consultation in Cameroon. Also, Venyuy et al. [12] looked at factors associated with the late start of late ANC among pregnant women in the Saint Elizabeth General Hospital Shisong (SEGHS), Cameroon, and found the cost of service, having a source of income, distance to a health facility, parity and level of education as determinants of late ANC in Cameroon [12]. The aforementioned studies were restricted to specific health facilities which present a challenge to understand determinants of early ANC attendance from a national scale. Therefore, there is a dearth of information on predictors that combined both the individual and community-level factors and the uptake of early ANC in Cameroon. Hence, the present study aimed at investigating the association between individual and community-level factors and early ANC uptake in Cameroon using nationally representative data. Findings of this study would be useful to the Ministry of Public Health and other agencies in Cameroon in programme designs targeted at early ANC uptake among women in their reproductive age group and also help in the attainment of SDG 3.1.

### Theoretical framework

Maternal healthcare utilisation is shaped by complex interwoven drivers including social, cultural, economic and religious factors [13, 14]. Therefore, for early ANC and its drivers which are at the core of this paper to be well-understood and for conceptual clarity, theoretical guidance is essential. Considering the primary focus of this study which seeks to investigate individual and community-level factors’ influence on early ANC, Anderson’s Behavioural Model (BM) of healthcare utilisation [15, 16] shall guide this paper. The BM explains that healthcare utilisation hinges on predisposing, enabling, and need factors operating at both individual and contextual domains [16, 17]. The predisposing factors are demographics and social structures whereas the enabling factors explain factors that facilitate individuals to use services including access to insurance, availability of resources such as income, access to free services, availability and access to the service [18]. Finally, the need factors are those that motivate service use including physical conditions, illness, or disease conditions [18]. The model provides a robust analytical framework for explaining drivers to maternal healthcare utilisation. In this study, it is anticipated that maternal early ANC utilisation shall be determined separately or jointly by individual-level factors including maternal age, wealth status, religion, education and marital status (predisposing factors) and community-level factors such as community literacy level and community socioeconomic status (enabling factors).

## Methods

### Data source

This study adopted a cross-sectional survey design. The data was extracted from the women’s file of the 2018 Cameroon Demographic and Health Survey (CDHS). The 2018 CDHS is the fifth Demographic and Health Survey conducted in Cameroon since its inception in 1991. It was designed to provide data for monitoring the population and health situation in Cameroon and to provide reliable estimates on demographics, maternal and other essential health issues [8]. The 2018 CDHS was implemented jointly by the National Institute of Statistics (NIS) and the Ministry of Public Health. Technical aid was provided by *The Centre Pasteur du Cameroun* (CPC) for HIV laboratory testing, the International Reference Centre Chantal Biya (IRCCB) provided quality control service whereas the ICF International provided technical assistance through the DHS Program [8].

The survey used multistage stratified/cluster sampling methods and identified a nationally representative sample of 13,773 eligible women for the individual women survey. However, a total of 13,527 women aged 15–49 completed the survey which yielded a response rate of 98%. Details on sampling, pre-testing, and other methodological issues can be obtained from the 2018 CDHS report [8]. The present study was restricted to 4,183 women aged 15–49 who had complete information on early ANC uptake and other selected variables of interest to the study.

### Description of study variables

#### Outcome variable

The outcome variable was early ANC. Early ANC is defined in the current study in line with the definition by the WHO [19, 20], thus seeking ANC service during the first trimester of gestation. In the 2018 CDHS women survey, women were asked “the timing for first antenatal check”. Those who indicated that their first ANC occurred from “zero to the third month” were considered as “early ANC” and those who had ANC visits “after the third month” were classified as “late ANC”. “Early ANC” was coded as “1” and “late ANC” was coded as “0”. For precision in responses, women who could not specify the time of initiating ANC were excluded from the analysis.

#### Explanatory variables

The present study considered 18 explanatory variables which are age, wealth status, religion, education, marital status, parity, occupation, partners’ education, frequency of reading newspaper/magazine, frequency of listening to radio, watching television, health decision-making capacity, getting medical help for self which includes getting permission to go, getting money needed for treatment, and distance to health facility (all captured as individual-level factors/predisposing factors). Residence, community literacy level, and community socioeconomic status were considered as community-level factors (enabling factors). These variables were selected owing to their influence on the uptake of ANC in other countries [20–22].

To make the results reader-friendly, religion of affiliation was recoded into “Christian”, “Islam”, “Animist”, “None” and “Other”; education was recoded into “no education”, “primary” and “secondary/higher”. Marital status was recoded into “married”, “cohabiting”. Considering the current fertility rate of Cameroon at 4.8 children per woman [8], parity was recoded into “one birth”, “two births”, “three births” and “four or more births”. Occupation was recoded into “not working” and “working”; partner’s education was recoded into “no education”, “primary” and “secondary/higher”; health decision-making capacity was recoded into “alone” and “not alone”; community literacy level was recoded into “low”, “medium” and “high”; and community socioeconomic status was recoded into “low”, “moderate” and “high”. In this study, community literacy level was calculated from the women who could read and write at all [23]. With regards to community socioeconomic status, it was measured as the percentage of households in the poorest quintile of Cameroon’s household wealth index [24].

### Analytical procedure

The analysis was done using Stata version 14.0. Firstly, the weighting factor inherent in the dataset (v005/100000) and the “svy command” were applied to cater for over and under sampling biases and to account for the complex survey design and generalizability of the findings respectively. Secondly, the proportion of women who booked early and late ANC was calculated. Furthermore, a univariate descriptive computation of both individual and community-level factors was done to display the summary statistics of the data. This was then followed by a bivariate descriptive analysis of early ANC across the individual and community-level factors and the results were in proportions and percentages. A chi-square test of independence was applied to assess the association between individual and community-level factors and early ANC and those with significant association were selected for the inferential analysis. The cut-off points for selecting a variable into the multilevel models were placed at 0.05 alpha threshold. Using the variance inflation factor (VIF) command, multicollinearity testing between the explanatory variables was done and the results indicated no evidence of multicollinearity between them (Mean VIF = 1.79, maximum VIF = 3.61, minimum VIF = 1.03).

At 95% confidence intervals (95% CIs), two-level multilevel logistic regression models were built to assess the association between the individual factors (predisposing factors) and community-level factors (enabling factors) and early ANC utilisation. The first was a null model (Model 0) to account for variability in early ANC which can be attributed to the clustering of the primary sampling units (PSUs)/clusters without the effect of both individual and community-level factors. The PSUs also referred to as clusters, contain the households. Subsequently, Model I considered individual-level factors solely, while Model II catered for only community-level factors and finally, Model III is the full model containing both individual and community-level factors. The results for the fixed effects were presented in adjusted odds ratio (aOR) and any odds less than one was interpreted as reduced likelihood of early ANC whilst any odds higher than 1 meant otherwise. Since the models were nested, the Akaike Information Criterion was used to measure their fitness [25]. The random effects which are measures of variation of early ANC visit across communities or clusters were expressed in terms of Intra-Class Correlation (ICC) and PSUs variance [25, 26]. These were calculated to quantify the degree of variation of early ANC booking across clusters and the proportion of variance explained by successive models. Specifically, the PSUs/cluster variance helped to determine the contribution of variables in each model to the remaining cluster variance.

### Ethical considerations

The study results, conclusions, and further recommendations were drawn from the 2018 CDHS which is an already existing dataset collected through the DHS Program in Cameroon. Therefore, this study required no ethical clearance. However, permission to download the dataset was requested from Measure DHS and the dataset was downloaded after the request was granted. The dataset is publicly accessible for download at https://dhsprogram.com/data/dataset/Cameroon_Standard-DHS_2018.cfm?flag=0. However, details on ethical considerations are provided by the 2018 CDHS report [8].

## Results

### Descriptive results for the study

The analysis indicated that, 46% (n = 1,931) [CI = 45.0–48.0] of the women aged 15–49 attended ANC earlier, with 54% (n = 2,252) [CI = 52.0–55.0] seeking ANC late (data not shown). Table 1 depicts the descriptive results of the study. The median age at which women started utilising early ANC was 28 (15 to 48) years. Generally, most of them were Christians (67%), married (75%), do not make health decisions alone (92%), belongs to the working class (75%), and do not read newspaper/magazine (83%) or listen to radio (63%) at all. Also, the majority of them declared that getting permission to seek medical help (68%), money needed for treatment (69%), and distance to health facility (61%) was not a problem when seeking medical help. A little over half of them (51%) resided in rural areas whereas 53% of them belong to a community of low socioeconomic status (Table 1).

**Table 1:** Individual and community-level factors and uptake of early ANC (Weighted N = 4,183).

Variable	Weighted (N)	Weighted (%)	Early ANC Uptake		COR	
			Late ANC (Weighted %)	Early ANC (Weighted %)		95% CI
Individual-level factors						
*Age (in years) (X*^*2*^ *= 16*.*1*, *p = 0*.*013)*						
Median age 28 (15, 48)						
15–19	327	8	63	37	Ref	1,1
20–24	825	20	52	48	1.52**	[1.17–1.97]
25–29	1146	27	54	46	1.50**	[1.17–1.93]
30–34	954	23	54	46	1.46**	[1.13–1.88]
35–39	639	15	51	49	1.54**	[1.17–2.02]
40–44	244	6	57	43	1.32	[0.95–1.84]
45–49	48	1	65	35	0.88	[0.49–1.60]
*Wealth status (X*^*2*^ *= 294*.*8*, *p<0*.*001)*						
Poorest	797	19	72	28	Ref	1,1
Poorer	893	21	63	37	1.59***	[1.28–1.97]
Middle	841	20	60	40	1.74***	[1.41–2.15]
Richer	868	21	46	54	2.83***	[2.29–3.51]
Richest	784	19	30	70	5.78***	[4.58–7.28]
*Religion (X*^*2*^ *= 108*.*4*, *p<0*.*001)*						
Christian	2815	67	48	52	Ref	1,1
Muslim	1243	30	69	31	0.48***	[0.41–0.55]
Animist	66	1.6	51	49	0.89	[0.52–1.53]
None	50	1.2	61	39	0.73	[0.42–1.28]
Other	9	0.2	35	65	3.85	[0.82–18.17]
*Education (X*^*2*^ *= 244*.*8*, *p<0*.*001)*						
No education	989	24	73	27	Ref	1,1
Primary	1341	32	59	41	1.79***	[1.48–2.16]
Secondary/ Higher	1853	44	41	59	3.66***	[3.06–4.37]
*Marital status (X*^*2*^ *= 13*.*9*, *p<0*.*001)*						
Married	3124	75	56	44	Ref	1,1
Cohabiting	1059	25	48	52	1.29***	[1.13–1.47]
*Parity (X*^*2*^ *= 48*.*7*, *p<0*.*001)*						
One birth	672	16	49	51	Ref	1,1
Two births	792	19	45	55	1.11	[0.91–1.37]
Three births	724	17	55	45	0.84	[0.68–1.04]
Four or more births	1995	48	60	40	0.66***	[0.55–0.78]
*Occupation (X*^*2*^ *= 0*.*5*, *p = 0*.*479)*						
Not working	1055	25	54	46	Ref	1,1
Working	3128	75	54	46	0.95	[0.83–1.09]
*Partner’s education (X*^*2*^ *= 222*.*81*, *p<0*.*001)*						
No education	814	20	74	26	Ref	1,1
Primary	1347	32	60	40	1.81***	[1.48–2.22]
Secondary/Higher	2022	48	43	57	3.62***	[2.99–4.38]
*Frequency of reading newspaper/magazine (X*^*2*^ *= 104*.*8*, *p<0*.*001)*						
Not at all	3484	83	58	42	Ref	1,1
Less than once a week	507	12	37	63	2.14***	[1.78–2.58]
At least once a week	192	5	29	71	2.89***	[2.12–3.95]
*Frequency of listening to radio (X*^*2*^ *= 71*.*7*, *p<0*.*001)*						
Not at all	2622	63	59	41	Ref	1,1
Less than once a week	827	20	49	51	1.48***	[1.27–1.73]
At least once a week	734	17	42	58	1.91***	[1.63–2.25]
*Frequency of watching television (X*^*2*^ *= 231*.*6*, *p<0*.*001)*						
Not at all	2000	48	67	33	Ref	1,1
Less than once a week	428	10	55	45	1.60***	[1.30–1.96]
At least once a week	1755	42	40	60	2.79***	[2.44–3.18]
*Getting medical help for self*: *getting permission to go (X*^*2*^ *= 8*.*0*, *p = 0*.*005)*						
Big problem	1351	32	58	42	Ref	1,1
Not a big problem	2832	68	53	47	1.20**	[1.06–1.37]
*Getting medical help for self*: *getting money needed for treatment (X*^*2*^ *= 44*.*5*, *p<0*.*001)*						
Big problem	2875	69	58	42	Ref	1,1
Not a big problem	1308	31	46	54	1.56***	[1.37–1.78]
*Getting medical help for self*: *distance to health facility (X*^*2*^ *= 44*.*2*, *p<0*.*001)*						
Big problem	1641	39	62	38	Ref	1,1
Not a big problem	2542	61	50	50	1.53***	[1.35–1.73]
*Health decision making capacity (X*^*2*^ *= 6*.*9*, *p = 0*.*009)*						
Alone	316	8	46	54	Ref	1,1
Not alone	3867	92	55	45	0.74**	[0.60–0.93]
Community-level factors						
*Residence (X*^*2*^ *= 98*.*5*, *p<0*.*001)*						
Urban	2032	49	45	55	Ref	1,1
Rural	2151	51	63	37	0.54***	[0.48–0.61]
*Community literacy level (X*^*2*^ *= 181*.*5*, *p<0*.*001)*						
Low	1595	38	67	33	Ref	1,1
Medium	1375	33	52	48	1.85***	[1.58–2.15]
High	1213	29	40	60	2.84***	[2.43–3.31]
*Community socioeconomic status (X*^*2*^ *= 198*.*5*, *p<0*.*001)*						
Low	2208	53	65	35	Ref	1,1
Moderate	569	13	54	46	1.48***	[1.24–1.77]
High	1406	34	38	62	2.69***	[0.54–0.64]

COR = Crude Odds Ratio, CI = Confidence Interval in square brackets; Ref = Reference Category

*p<0.05

**p<0.01

***p<0.001.

Source: Deduced from 2018 CDHS.

The analysis revealed that 49% of women aged 35–39 sought early ANC. Early ANC utilisation was phenomenal among the richest (70%), affiliates of other religions (65%), women with secondary or higher education (59%), and those cohabiting (52%). Early ANC utilisation peaked among those at parity two (55%). Those whose partners had secondary or higher education (57%) were the highest to utilise early ANC, just as among those that read newspaper/magazine at least once a week (71%) (Table 1).

Early ANC was high among women who listened to radio at least once a week (58%) and those who watched television at least once a week (60%). Similarly, early ANC was phenomenal among women who indicated that it was not a big problem (47%) to seek permission for healthcare and for those women who indicated that obtaining money for treatment is not a problem (54%). Furthermore, 50% of women who declared that distance to health facility was unproblematic as well as and 54% of women who made health decisions alone sought early ANC. A significant proportion of urban residents (55%), communities with high literacy (60%) and socioeconomic status (62%) had early ANC (Table 1).

#### Fixed effect results

The third model (Model III) in Table 2 shows the fixed effect results of individual and community-level factors on early ANC. Compared with women aged 15–19, the odds to seek early ANC increased as one advance in age, especially among those aged 35–39 [aOR = 1.78, CI = 1.24–2.57]. The richest were about two-fold probable to have early ANC compared with the poorest [aOR = 2.43, CI = 1.63–3.64]. Muslims had lesser odds to seek early ANC compared with Christians [aOR = 0.73, CI = 0.60–0.88]. Those with secondary/higher education had higher odds to seek early ANC compared with those without formal education [aOR = 1.38, CI = 1.05–1.82].

**Table 2:** A mixed effect of individual and community-level factors on early initiation of antenatal care.

Independent variables	Model 0	Model I	Model II	Model III
	aOR[95%CI]	aOR[95%CI]	aOR[95%CI]	aOR[95%CI]
Fixed effect results				
Individual-level factors				
*Age*				
15–19		Ref		Ref
20–24		1.41*[1.05–1.89]		1.41*[1.04–1.89]
25–29		1.47*[1.08–2.01]		1.47*[1.08–2.00]
30–34		1.50*[1.07–2.10]		1.50*[1.07–2.10]
35–39		1.79**[1.24–2.57]		1.78**[1.24–2.57]
40–44		1.59*[1.05–2.43]		1.60*[1.05–2.44]
45–49		1.33[0.67–2.63]		1.34[0.67–2.63]
*Wealth status*				
Poorest		Ref		Ref
Poorer		1.31*[1.02–1.69]		1.32*[1.02–1.71]
Middle		1.23[0.94–1.61]		1.27[0.95–1.69]
Richer		1.60**[1.18–2.18]		1.65**[1.16–2.34]
Richest		2.45***[1.73–3.47]		2.43***[1.63–3.64]
*Religion*				
Christian		Ref		Ref
Muslim		0.72**[0.58–0.87]		0.73**[0.60–0.88]
Animist		1.72[0.93–3.17]		1.73[0.94–3.21]
None		0.91[0.49–1.68]		0.91[0.49–1.67]
Other		2.45[0.58–14.55]		2.93[0.58–14.72]
*Education*				
No education		Ref		Ref
Primary		1.20[0.94–1.53]		1.20[0.94–1.53]
Secondary/Higher		1.40**[1.06–1.82]		1.38*[1.05–1.82]
*Marital status*				
Married		Ref		Ref
Cohabiting		0.98[0.83–1.15]		0.97[0.82–1.15]
*Parity*				
One birth		Ref		Ref
Two births		0.91[0.72–1.16]		0.91[0.72–1.16]
Three births		0.66**[0.51–0.85]		0.66**[0.51–0.86]
Four or more births		0.63**[0.49–0.82]		0.63**[0.49–0.82]
*Partner’s education*				
No education		Ref		Ref
Primary		1.18[0.91–1.51]		1.18[0.92–1.51]
Secondary/Higher		1.52**[1.17–1.98]		1.52**[1.17–1.98]
*Frequency of reading newspaper/magazine*				
Not at all		Ref		Ref
Less than once a week		1.28*[1.03–1.59]		1.28*[1.03–1.59]
At least once a week		1.24[0.87–1.78]		1.25[0.87–1.79]
*Frequency of listening to radio*				
Not at all		Ref		Ref
Less than once a week		0.95[0.78–1.14]		0.94[0.78–1.14]
At least once a week		1.05[0.86–1.28]		1.05[0.85–1.28]
*Frequency of watching television*				
Not at all		Ref		Ref
Less than once a week		1.06[0.83–1.37]		1.06[0.82–1.37]
At least once a week		1.23[0.99–1.54]		1.23[0.98–1.53]
*Getting medical help for self*: *getting permission to go*				
Big problem		Ref		Ref
Not a big problem		0.99[0.83–1.17]		0.98[0.83–1.16]
*Getting medical help for self*: *getting money needed for treatment*				
Big problem		Ref		Ref
Not a big problem		1.04[0.88–1.24]		1.04[0.88–1.24]
*Getting medical help for self*: *distance to health facility*				
Big problem		Ref		Ref
Not a big problem		1.17[0.99–1.39]		1.18[1.00–1.40]
*Health decision making capacity*				
Alone		Ref		Ref
Not alone		0.95[0.74–1.22]		0.95[0.74–1.22]
Community-level factors				
*Residence*				
Urban			Ref	Ref
Rural			0.90[0.73–1.11]	1.13[0.91–1.41]
*Community literacy level*				
Low			Ref	Ref
Medium			1.57***[1.28–1.94]	0.97[0.77–1.22]
High			2.00***[1.59–2.52]	1.05[0.81–1.36]
*Community socioeconomic status*				
Low			Ref	Ref
Moderate			1.15[0.88–1.49]	0.94[0.73–1.22]
High			1.92***[1.49–2.47]	1.13[0.86–1.48]
Random effect results				
PSU/Cluster Variance[95%CI]	0.53[0.40–0.72]	0.16[0.09–0.29]	0.22[0.14–0.35]	0.16[0.09–0.29]
ICC	0.14	0.05	0.06	0.05
LR Test	141.55***	18.43***	34.29***	17.11***
Wald X^2^	Ref	344.18***	168.02***	347.85***
Model specification diagnosis				
Log likelihood	-2816.3	-2644.2	-2741.7	-2642.5
AIC	5636.7	5356.4	5497.5	5362.9
BIC	5649.3	5571.9	5541.9	5610.1

aOR = adjusted Odds Ratio, CI = Confidence Interval in square brackets; Ref = Reference Category

*p<0.05

**p<0.01

***p<0.001.

Relative to women at parity one, women at parity four or more had lesser odds to have early ANC [aOR = 0.63, CI = 0.49–0.82]. The findings showed that women whose partners had secondary or higher education had a higher likelihood to utilise early ANC compared with those whose partners had no education [aOR = 1.52, CI = 1.17–1.98]. The odds to seek early ANC increased among women who read newspaper/magazine less than once a week compared with their counterparts who were not reading newspaper/magazine at all [aOR = 1.28, CI = 1.03–1.59].

### Random effect results

From Table 2, the random effect results indicated that there was variation in early ANC utilisation [σ^2^ = 0.53, CI = 0.40–0.72]. Specifically, about 14% variance in early ANC is attributable to variation in the intra-class correlation (ICC) features (ICC = 0.14). The ICC values were fairly stable in Model I (ICC = 0.05), Model II (ICC = 0.06), and in Model III (ICC = 0.05).

From the model fit test, it was found that the ideal value of the estimated coefficient of the null model was lower (log likelihood = -2816.3), but it improved in the succeeding models, particularly in model III (log likelihood = -2642.5). Similarly, the null model was less appropriate (Akaike Information Criteria [AIC] = 5636.7). However, there was a substantial improvement in the desirability of the models specifically in model III (AIC = 5362.915). Therefore, Model III emerged as the most suitable model.

## Discussion

This study investigated the effects of individual and community-level factors on early ANC in Cameroon. The present study recorded an early ANC prevalence of 46% among women aged 15–49 in Cameroon. This is below the WHO recommendation [27] and what was reported in Ghana [20]. The probable reason could be due to differences in the population characteristics surveyed among the two countries. This calls for strong political will from the Government of Cameroon, utilising the Ministry of Public Health and other agencies, to institute measures that could minimise the challenges obstructing women’s access to early ANC uptake.

The odds to utilise early ANC services increased across ages, especially among those aged 35–39 relative to those aged 15–19. This is in line with the theoretical proposition that demographic characteristics influence maternal healthcare utilisation [15, 16, 18]. A recent study from Ghana also found that there were increasing odds for mothers aged 20 and above for first-time mothers to initiate ANC visits in the first trimester of pregnancy compared to mothers below age 20 [20]. A plausible reason accounting for this observation is that most societies discourage pregnancy at teenage and in extreme instances, teenagers who get pregnant are dismissed from school and stigmatised. Hence some teenagers tend to be reluctant to disclose their pregnancy status and fail to utilise ANC services to avoid social pressures and unfair treatment.

The richest had over two-fold likelihood to seek early ANC service which affirms the theoretical stands that maternal wealth predicts their healthcare utilisation [15, 16, 18]. This is consistent with findings by Gebremeskel et al. [27] which indicated that monthly low-income level was significantly associated with late starting of first ANC in Ethiopia. Manyeh et al. [20] also found that there was an increased odds of women who belong to the richest socioeconomic status to initiate ANC visits in the first trimester compared with the poorest in Ghana. Similarly, Tolefac et al. [11] also observed that the odds of starting first ANC late were 3 times higher if the woman had a monthly income of less than 200 US dollars in Cameroon. The results are not surprising since financial constraints have been observed as a key challenge obstructing women’s access to early ANC services in sub-Sahara Africa [9, 28, 29] and the same reason could account for the observed phenomenon in this study. This connotes that families in Cameroon should financially assist women especially the poor so that they can afford user-fees associated with the utilisation of early ANC.

The present study noted that the probability to utilise early ANC declined among the Muslim compared with Christians. Kuuire et al. [30] also found that women who were Muslims and traditionalists were less likely to initiate ANC in the first trimester in Malawai and Nigeria. Apart from that, studies have reported mixed results concerning the influence of religion on ANC attendance. In Nigeria [31, 32] and Zimbabwe [33], it was known that Christians were more likely to use ANC service compared with traditional African religion and Muslims and a similar finding was reported in Benin [34]. Findings from Ghana [35] and Kenya [36] also established that women who had no religion were less likely to attend ANC. Perhaps, the variance in religious teachings could explain the observed difference in early ANC uptake among women in Cameroon. However, the substantive evidence on religion and ANC utilisation suggests religion influences ANC uptake; hence, a further study including a qualitative type, to explore the influence of religion on early uptake of ANC services is worthy to understand the phenomenon better.

Those with secondary/higher education had higher odds to seek early ANC compared with those without education just as among women whose partners had completed secondary or higher education compared with those whose partners had no education. Theoretically, education empowers women to be able to make informed decisions concerning their health and hence seek healthcare [15, 16, 18]. In Ethiopia, Gebresilassie et al. [37] also found that mothers who have completed secondary school and higher were two-fold probable to seek timely ANC services compared to the mothers who completed primary school or had no formal education. The authors explained that educated mothers might be knowledgeable about pregnancy issues, the benefits of ANC, and early booking and hence, tend to book ANC services on time. Another plausible reason in explaining the relationship between education and ANC service utilisation is that education influences the chances to better employment opportunities rendering the elites financially independent, and at the same time, education propels women to be better informed on the significance of using early ANC [38]. Based on these, it is possible women from Cameroon with higher education or those with elite partners shall be inclined to early ANC. The results are consistent with several studies that indicated that educated women and those whose partners possess higher education are more likely to use antenatal services and also initiate this within the first trimester of pregnancy [39–41].

Relative with those at parity one, women at parity four or more had lesser odds to seek early ANC service. This confirms the theoretical proposition that parity as a predisposing factor, plausibly influences maternal healthcare utilisation [15–17]. Parity has been observed in several studies as a determinant of ANC initiation with those at low or zero parity seeking early bookings whereas their counterparts with higher parity tend to initiate ANC late [30, 42–46]. Two arguments have been advanced in explaining the role of parity on ANC utilisation. Some scholars argue that women, especially those at higher parity who have had previous pregnancies, may consider themselves as experienced and are often accustomed to the routine care given to women during ANC, hence delay in seeking ANC earlier or reducing the number of ANC visits [47]. Others also hold that the decline in ANC utilisation among women of higher parity is as a result of inadequate resources in the family and previous exposure to poor treatment received from health personnel [39]. Any of these explanations could account for the reason why women of low parity had a higher likelihood to seek early ANC services relative to those of higher parity.

The likelihood to seek early ANC increased among those that read newspaper/magazine less than once a week compared with their counterparts that do not read newspaper/magazine. This finding is in line with a study by Geta and Yallew [21] which showed that Ethiopian mothers who had access to TV/radio were twice more likely to initiate early ANC than those who were not. In Nepal, Acharya et al. [48] also find out that mothers exposed to mass media were more likely to be attending ANC visits than mothers who were not exposed. Similarly, Edward [49] observed that women having access to media were more likely to use antenatal care services relative to those with no access in Uganda. Theoretically, it is possible for women in Cameroon who are exposed to newspaper/magazine to be inclined to early ANC since some of the newspapers/magazines convey health information and benefits of seeking ANC services which end up enlightening its readers [15–17].

Finally, the variance in the propensity to seek early ANC was due to variance in the primary sampling units (PSUs)/clusters. This is suggestive that in designing a health information and sensitisation programs targeted at early ANC uptake, attention should be paid to the cluster variations in Cameroon.

### Strength and limitations of the study

This study is novel since it presented a current view of individual and community-level factors that influence early ANC utilisation in Cameroon. Also, the study made use of a nationally representative survey with a relatively large sample size (N = 4,183). It also applied rigorous and advanced analytical methods to analyse the data for the study. However, the CDHS dataset was generated from the respondents’ self-report and previous ANC experiences, including time of ANC initiation, therefore recall bias is inevitable. Also, the study is liable to social desirability biases. Again, the cross-sectional nature of the survey suggests that the current study only measured an association between individual and community level factors and early ANC utilisation and failed to establish a causal relationship between early ANC and individual and community-level factors.

## Conclusion

The present study noted that there was a low uptake of early ANC in Cameroon. The study further revealed that maternal age, wealth status, religion of affiliation, educational attainment, partners’ educational attainment, parity, and access to newspaper/magazine (all are individual-level factors) predicted early ANC uptake in Cameroon. The low uptake of early ANC in Cameroon suggests that the Government of Cameroon through its Ministry of Public Health should institute proactive interventions to address challenges that prevent women’s access to early ANC. The Departments of Health Promotion, Health Information Center and eHealth under the Ministry of Public Health, Cameroon, have to strengthen mass sensitisation programs on early ANC uptake and such programs should consider individual differences such as age, wealth status, education, and religious affiliation, in its program design. Such programs should also employ mass media, especially newspaper/magazine in reaching out to women in Cameroon.

## References

[1] United Nations (UN). Sustainable development goals. New York: United Nations Department of Economic and Social Affairs. https://sustainabledevelopment.un.org/index.html. 2015.

[2] World Health Organisation (WHO). Strategies toward ending preventable maternal mortality (EPMM). Geneva: World Health Organization; 2015.

[3] Graham W, Woodd S, Byass P, Filippi V, Gon G, Virgo S, et al. Diversity and divergence: the dynamic burden of poor maternal health. *Lancet (London*, *England)*, 388(10056), 2016; 2164–2175. doi: 10.1016/S0140-6736(16)31533-1 27642022

[4] Moller AB, Petzold M, Chou D, Say L. Early antenatal care visit: a systematic analysis of regional and global levels and trends of coverage from 1990 to 2013. *Lancet Glob Health*, 2017; 5: e977–83. doi: 10.1016/S2214-109X(17)30325-X 28911763 PMC5603717

[5] Villar J, Ba’aqeel H, Piaggio G, Lumbiganon P, Miguel Belizán J, Farnot U, et al. WHO antenatal care randomised trial for the evaluation of a new model of routine antenatal care. *Lancet (London*, *England)*, 357(9268), 2001; 1551–1564. doi: 10.1016/s0140-6736(00)04722-x 11377642

[6] WHO. WHO antenatal care randomized trial: manual for the implementation of the new model; 2002.

[7] Zolotor AJ, Carlough MC. Update on prenatal care. *Am Fam Physician*, 2014; 89: 199–208. 24506122

[8] National Institute of Statistics (INS) and ICF. Cameroon Demographic and Health Survey 2018. Yaoundé, Cameroon and Rockville, Maryland, USA: INS and ICF;2020.

[9] Halle-Ekane GE, Obinchemti TE, Nzang JL, Mokube NM, Njie MM, Njamen TN, et al. Assessment of the content and utilization of antenatal Care Services in a Rural Community in Cameroon: a cross-sectional study. *Open J Obstet & Gynecol*, 2014; 29;4(14):846.

[10] Halle-Ekane GE, Fotabong CM, Njotang PN, Atashili J, Bechem NN, Nsagha DS, et al. Quality of antenatal care and outcome of pregnancy in a semi-urban area in Fako Division, Cameroon: a cross-sectional study. *Women Health Open J*. 2015; 1(2): 31–39. doi: 10.17140/WHOJ-1-105

[11] Tolefac PN, Halle-Ekane GE, Agbor VN, Sama CB, Ngwasiri C, Tebeu PM. Why do pregnant women present late for their first antenatal care consultation in Cameroon? *Maternal Health*, *Neonatology*, *and Perinatology*, 2017; 2–6. doi: 10.1186/s40748-017-0067-8 29255616 PMC5727881

[12] Venyuy MA, Cumber SN, Nkfusai CN, Bede F, Ijang YP, Wepngong E, et al. Determinants to late antenatal clinic start among pregnant women: the case of Saint Elizabeth General Hospital, Shisong, Cameroon. *Pan African Medical Journal*, 2020; 35:112. doi: 10.11604/pamj.2020.35.112.18712 32637010 PMC7321684

[13] Amin R, Shah NM, Becker S. Socioeconomic factors differentiating maternal and child health-seeking behaviour in rural Bangladesh: A cross-sectional analysis. Int J Equity Health. 2010;9:9. doi: 10.1186/1475-9276-9-9 20361875 PMC2859349

[14] Lindelow M. The utilisation of curative healthcare in Mozambique: does income matter? J Afr Econ. 2016;14(3):435–82.

[15] Andersen R, Newman JF. Societal and individual determinants of medical care utilization in the United States. Milbank Mem Fund Q Health Soc. 1973; 51(1):95–124. 4198894

[16] Andersen RM. National health surveys and the behavioral model of health services use. Med Care. 2008;46(7):647–53. doi: 10.1097/MLR.0b013e31817a835d 18580382

[17] Babitsch B, Gohl D, Von Lengerke T. Re-revisiting Andersen’s Behavioral Model of Health Services Use: A systematic review of studies from 1998 to 2011. GMS Psycho-Social-Medicine. 2012;9:1–15. 10.3205/psm000089 PMC3488807 23133505

[18] Azfredrick EC. Using Anderson’s model of health service utilization to examine use of services by adolescent girls in southeastern Nigeria, International Journal of Adolescence and Youth, 2016; 21:4, 523–529. 10.1080/02673843.2015.1124790.

[19] WHO. WHO Recommendations on Antenatal Care for a Positive Pregnancy Experience: Summary. Geneva: WHO; 2018. 28079998

[20] Manyeh AK, Amu A, Williams J, Gyapong M. Factors associated with the timing of antenatal clinic attendance among first time mothers in rural southern Ghana. BMC Pregnancy and Childbirth, 2020;20:47, 2–7. doi: 10.1186/s12884-020-2738-0 31959137 PMC6972022

[21] Geta MB, Yallew WW. Early Initiation of Antenatal Care and Factors Associated with Early Antenatal Care Initiation at Health Facilities in Southern Ethiopia. *Advances in Public Health*; 2017, Article ID 1624245, 1–6, 10.1155/2017/1624245.

[22] Teshale AB, Tesema GA. Prevalence and associated factors of delayed first antenatal care booking among reproductive age women in Ethiopia; a multilevel analysis of EDHS 2016 data. *PLoS ONE*, 2020;15(7): e0235538. doi: 10.1371/journal.pone.0235538 32628700 PMC7337309

[23] Zegeye B, Olorunsaiye CZ, Ahinkorah BO, Ameyaw EK, Budu E, Seidu AA, et al. Individual/Household and Community-Level Factors Associated with Child Marriage in Mali: Evidence from Demographic and Health Survey. *BioMed Research International*, 2021, 5529375. doi: 10.1155/2021/5529375 34239924 PMC8241519

[24] Janevic T, Sarah PW, Leyla I, Elizabeth BH. Individual and community level socioeconomic inequalities in contraceptive use in 10 Newly Independent States: a multilevel cross-sectional analysis. *International Journal for Equity in Health*, 2012; 11(1), 1–13. doi: 10.1186/1475-9276-11-69 23158261 PMC3520858

[25] Ahinkorah BO. Individual and contextual factors associated with mistimed and unwanted pregnancies among adolescent girls and young women in selected high fertility countries in sub-Saharan Africa: A multilevel mixed effects analysis. *PLoS ONE*, 2020;15(10): e0241050. doi: 10.1371/journal.pone.0241050 33091050 PMC7580885

[26] Solanke BL, Oyinlola FF, Oyeleye OJ, Ilesanmi BB. Maternal and community factors associated with unmet contraceptive need among childbearing women in Northern Nigeria. *Contraception and reproductive medicine*, 2019;4(1):11. doi: 10.1186/s40834-019-0093-1 31497311 PMC6717978

[27] Gebremeskel F, Dibaba Y, Admassu B. Timing of first antenatal care attendance and associated factors among pregnant women in Arba Minch town and Arba Minch District, Gamo Gofa zone, South Ethiopia. *J Environ Public Health*; 2015:e971506. doi: 10.1155/2015/971506 26543485 PMC4620253

[28] Gross K, Alba S, Glass TR, Schellenberg JA, Obrist B. Timing of antenatal care for adolescent and adult pregnant women in south-eastern Tanzania. *BMC Preg & childbirth*, 2012;12(1):16. doi: 10.1186/1471-2393-12-16 22436344 PMC3384460

[29] Andrew EV, Pell C, Angwin A, Auwun A, Daniels J, Mueller I, et al. Factors affecting attendance at and timing of formal antenatal care: results from a qualitative study in Madang, Papua New Guinea. *PloS one*, 2014;19;9(5): e93025. doi: 10.1371/journal.pone.0093025 24842484 PMC4026245

[30] Kuuire VZ, Kangmennaang J, Atuoye KN, Antabe R, Boamah SA, Vercillo S, et al. Timing and utilisation of antenatal care service in Nigeria and Malawi. *Global public health*, 2017;12(6), 711–727. doi: 10.1080/17441692.2017.1316413 28441926

[31] Ononokpono DN. Maternal health care in Nigeria: do community factors moderate the effects of individual-level education and ethnic origin? *African Population Studies*, 2015;29:1554–69.

[32] Adewuyi EO, Auta A, Khanal V, Bamidele OD, Akuoko CP, Adefemi K, et al. Prevalence and factors associated with underutilization of antenatal care services in Nigeria: a comparative study of rural and urban residences based on the 2013 Nigeria demographic and health survey. *PLoS One*, 2018;13:e0197324. doi: 10.1371/journal.pone.0197324 29782511 PMC5962076

[33] Makate M, Makate C. Prenatal care utilization in Zimbabwe: examining the role of community-level factors. *J Epidemiol Glob Health*, 2017;7:255–62. doi: 10.1016/j.jegh.2017.08.005 29110866 PMC7384567

[34] Yaya S, Uthman OA, Amouzou A, Ekholuenetale M, Bishwajit G. Inequalities in maternal health care utilization in Benin: a population based cross-sectional study. *BMC pregnancy and childbirth*, 2018;18(1), 194. doi: 10.1186/s12884-018-1846-6 29855277 PMC5984297

[35] Abor PA, Abekah-Nkrumah G, Sakyi K, Adjasi CKD, Abor J. The socioeconomic determinants of maternal health care utilization in Ghana. *Int J Soc Econ*, 2011;38:628–48.

[36] Banke-Thomas A, Banke-Thomas O, Kivuvani M, Ameh C. Maternal health services utilisation by Kenyan adolescent mothers: analysis of the demographic health survey 2014. *Sex Reprod Healthc*. 2017;12:37–46. doi: 10.1016/j.srhc.2017.02.004 28477930

[37] Gebresilassie B, Belete T, Tilahun W, Berhane B, Gebresilassie S. Timing of first antenatal care attendance and associated factors among pregnant women in public health institutions of Axum town, Tigray, Ethiopia, 2017: a mixed design study. *BMC Pregnancy and Childbirth*, 2019;19:340, 2–11, doi: 10.1186/s12884-019-2490-5 31533657 PMC6751589

[38] Grown C, Gupta GR, Pande R. Taking action to improve women’s health through gender equality and women’s empowerment. *The Lancet*, 2005;365:541–3. doi: 10.1016/S0140-6736(05)17872-6 15705464

[39] Simkhada B, Teijlingen ER, Porter M, Simkhada P. Factors affecting the utilization of antenatal care in developing countries: systematic review of the literature. *Journal of advanced nursing*, 61(3), 2008;244–260. doi: 10.1111/j.1365-2648.2007.04532.x 18197860

[40] Say L, Raine R. A systematic review of inequalities in the use of maternal health care in developing countries: examining the scale of the problem and the importance of context. *Bull World Health Organ*, 2007;85:812–9. doi: 10.2471/blt.06.035659 18038064 PMC2636485

[41] Tekelab T, Chojenta C, Smith R, Loxton D. Factors affecting utilization of antenatal care in Ethiopia: A systematic review and meta-analysis. *PLoS ONE*, 2019;14(4): e0214848. doi: 10.1371/journal.pone.0214848 30973889 PMC6459485

[42] Verney A, Reed BA, Lumumba JB. Factors associated with socio-demographic characteristics and antenatal care and iron supplement use in Ethiopia, Kenya, and Senegal. *Matern Child Nutr*, 2018;14:e12565. 10.1111/mcn.12565 PMC6866103 29493903

[43] Manzi A, Munyaneza F, Mujawase F, Banamwana L, Sayinzoga F, Thomson DR, et al. Assessing predictors of delayed antenatal care visits in Rwanda: a secondary analysis of Rwanda demographic and health survey 2010. *BMC Pregnancy Childbirth* 2014;14:290. doi: 10.1186/1471-2393-14-290 25163525 PMC4152595

[44] Ochako R, Fotso J-C, Ikamari L, Khasakhala A. Utilization of maternal health services among young women in Kenya: insights from the Kenya demographic and health survey, 2003. *BMC Pregnancy Childbirth*, 2011;11:1. doi: 10.1186/1471-2393-11-1 21214960 PMC3022772

[45] Oladokun A, Oladokun RE, Morhason-Bello I, Bello AF, Adedokun B. Proximate predictors of early antenatal registration among Nigerian pregnant women. *Ann Afr Med*, 2010;9:222–5. doi: 10.4103/1596-3519.70959 20935421

[46] Zegeye AM, Bitew BD, Koye DN. Prevalence and determinants of early antenatal care visit among pregnant women attending antenatal care in Debre Berhan health institutions, central Ethiopia. *Afr J Reprod Health*, 2013;17:130–6. 24558789

[47] Pell C, Meñaca A, Were F, Afrah NA, Chatio S, Manda-Taylor L, et al. Factors affecting antenatal care attendance: results from qualitative studies in Ghana, Kenya and Malawi. *PloS one*, 2013;8(1), e53747. doi: 10.1371/journal.pone.0053747 23335973 PMC3546008

[48] Acharya D, Khanal V, Singh JK, Adhikari M, Gautam S. Impact of mass media on the utilization of antenatal care services among women of rural community in Nepal *BMC Res Notes*, 2015;8:345, 2–6, doi: 10.1186/s13104-015-1312-8 26264412 PMC4534014

[49] Edward B. Factors influencing the utilisation of antenatal care content in Uganda. *Australas Med J*., 2011;4(9):516. doi: 10.4066/AMJ.2011.849 23393544 PMC3562912

